# Bovine Serum Albumin Enhances the Quantitative Performance of Polydimethylsiloxane-Based Chamber Digital PCR by Suppressing Surface Adsorption

**DOI:** 10.3390/mi17070791

**Published:** 2026-06-28

**Authors:** Eri Tsunoi, Kazuo Hosokawa, Hitoshi Ohmori, Kae Sato

**Affiliations:** 1 Department of Chemical and Biological Sciences, Faculty of Science, Japan Women’s University, 2-8-1 Mejirodai, Bunkyo, Tokyo 112-8681, Japan; 2 Materials Fabrication Laboratory, RIKEN Pioneering Research Institute, 2-1 Hirosawa, Wako, Saitama 351-0198, Japan

**Keywords:** digital PCR, PDMS device, nonspecific adsorption, bovine serum albumin, chamber-based dPCR

## Abstract

Polydimethylsiloxane (PDMS) surfaces are highly hydrophobic, and non-specific biomolecule adsorption is a well-known limitation in microfluidic PCR systems. Flow-based microfluidic PCR has been extensively studied, but the impact of surface adsorption on quantitative performance in closed-chamber digital PCR (dPCR) platforms remains poorly characterized. This adsorption may reduce the effective concentrations of key reaction components and compromise quantification accuracy. Therefore, in this study, we evaluated two approaches to prevent molecular adsorption in PDMS-based cdPCR systems: (i) the addition of chemical additives to the PCR reaction mixture and (ii) the incorporation of hydrophilizing agents into PDMS, with solution-phase additives proving more effective in this system. We investigated the effects of the reaction additives bovine serum albumin (BSA), Blocking One-P, and dextran on DNA quantification using a PDMS-based dPCR chip. A single-concentration comparison showed that 1.1% BSA produced the highest average DNA copy number (0.091 ± 0.010 copies/well), compared to the no-additive condition (0.039 ± 0.010 copies/well), corresponding to an approximately 2.3-fold increase, whereas Blocking One-P and dextran had no substantial effects. Dilution series experiments were then conducted under BSA-added and BSA-free conditions using plasmid DNA and cDNA derived from HSC4 cells as templates. In both cases, BSA improved quantitative linearity, as reflected by the increased slopes and coefficients of determination.

## 1. Introduction

Digital PCR (dPCR) has emerged as a powerful technique for absolute nucleic acid quantification, enabling high sensitivity and precision without the need for calibration curves [1,2]. Among the various dPCR implementations, chamber- and chip-based dPCR (cdPCR) platforms [1,2,3,4,5] have attracted considerable attention owing to their potential compatibility with standard laboratory equipment, such as conventional thermal cyclers and fluorescence microscopes.

Hosokawa et al. developed a representative cdPCR system based on a simple microfluidic chip, demonstrating that highly sensitive digital quantification can be achieved without the need for expensive, dedicated instrumentation [4,5]. Consequently, cdPCR provides a practical route to expand dPCR accessibility to a wide range of laboratories.

Polydimethylsiloxane (PDMS) is employed as the device material in many cdPCR systems [4,5,6,7,8,9,10,11] because of its low cost, ease of fabrication, and compatibility with microstructuring processes [12]. PDMS is a silicone elastomer that exhibits hydrophobicity and high permeability to small molecules, water, and gases [12,13]. These material properties make PDMS attractive for rapid prototyping, but also raise concerns regarding its interaction with the biochemical reagents used in PCR.

Indeed, Gökaltun et al. and others have reported that biomolecules and PCR components can be adsorbed to PDMS surfaces, potentially limiting the use of PDMS-based microdevices [14,15]. This adsorption may reduce the effective concentration of essential reaction components, including enzymes, primers, and nucleic acids, thereby affecting PCR performance [15]. While PDMS-related adsorption has been widely recognized in microfluidic PCR systems, most previous studies have focused on flow-based microfluidics or qualitative PCR performance, rather than on quantitative outcomes in sealed chamber-based dPCR platforms.

Furthermore, existing mitigation strategies for PDMS adsorption typically rely on surface modification techniques, such as plasma treatment, chemical coatings, or the use of alternative materials [9,12,14]. Although effective, these approaches often introduce additional fabrication complexity, stability issues, or specialized equipment requirements, potentially compromising the simplicity and accessibility that constitute the key advantages of cdPCR systems operated with standard laboratory instruments.

In contrast, modifying the reaction solution by introducing additives to block PDMS represents a practical and device-independent strategy [15,16]. However, the extent to which such solution-phase additives affect absolute quantification in chamber-based dPCR systems has not been systematically evaluated. Particularly, how these additives affect copy-number readouts under experimental conditions compatible with conventional thermal cyclers and fluorescence imaging remains unclear.

Therefore, in this study, we evaluated two approaches to prevent molecular adsorption in PDMS-based cdPCR systems: (i) the addition of chemical additives to the PCR reaction mixture and (ii) the incorporation of hydrophilizing agents into PDMS, with a particular focus on the effectiveness of solution-phase additives. By systematically evaluating their influence on absolute copy-number determination using both plasmid DNA and cDNA templates, and by comparing the results with conventional real-time PCR measurements, we aimed to develop practical strategies for quantitative accuracy improvement in PDMS-based cdPCR platforms.

## 2. Materials and Methods

### 2.1. Fabrication of PDMS Microwell Array (MWA) Chip

PDMS-based microwell chips for dPCR were fabricated based on the chip design reported in previous studies [4,5] using the same fundamental microstructure. Each chip consisted of square microwells with lateral dimensions of 50 µm × 50 µm and a depth of 100 µm. The microwells were arranged in an array of 46 rows and 60 columns, yielding 2760 microwells per unit. Nine of these units were arranged in a 3 × 3 configuration, resulting in a total of 24,840 microwells per chip.

Two types of PDMS MWA chips were prepared to evaluate the effect of surface properties: with or without an internal hydrophilizing agent. PDMS MWA chips without an internal hydrophilizing agent were prepared by mixing the PDMS base and curing agent (SILPOT 184, Dow Corning, Midland, MI, USA) at a mass ratio of 10:1, followed by molding using a replica molding process.

A blend-type PDMS hydrophilizing agent (MMS-003, 059-04, Arakawa Chemical Industries, Osaka, Japan) was added to the uncured PDMS mixture for the hydrophilized PDMS MWA chip. Specifically, the hydrophilizing agent was incorporated at 8.0% (*w*/*w*) relative to the total mass of the PDMS prepolymer (PDMS base + curing agent), which was mixed in a ratio of 10:1. The mixture was then molded using the replica molding procedure. For both chip types, the molded PDMS prepolymers were thermally cured by baking at 55 °C for 16 h.

### 2.2. Plasmid DNA as a dPCR Template

Plasmid DNA containing the SARS-CoV-2 N-gene sequence (SARS-CoV-2 Positive Control, *N* gene; N2117S, New England Biolabs, Ipswich, MA, USA) was used as the DNA template. Plasmid DNA was diluted with EASY Dilution II (Takara Bio, Siga, Japan) to a concentration of 4.0 × 10^4^ copies/µL. This diluted solution was added to the reaction mixture to achieve a final concentration of 1.0 × 10^3^ copies/µL in the PCR. Based on the well volume and partition number of the dPCR chip used in this study, this condition was designed to yield a calculated average DNA copy number of 0.25 copies/well during dPCR analysis.

The PCR reaction mixture consisted of 1.0 µL of the SARS-CoV-2 positive control solution, 20 µL of QuantStudio 3D Digital PCR Master Mix v2 (A26358, Thermo Fisher Scientific, Waltham, MA, USA), and 8 µL of NIID_2019-nCoV_N Primer/Probe Mix (XD0008, Takara Bio). As QuantStudio 3D Digital PCR Master Mix v2 does not contain ROX dye, 0.8 µL of 50× ROX Dye (high ROX, KAPA Biosystems, Wilmington, MA, USA) was added as an external reference dye to confirm proper partitioning of the reaction mixture. RNase-free water and additives were added as needed to adjust the final reaction volume to 40 µL. The primer and probe sequences used in this study are listed in Table 1. The expected length of the PCR amplicon was 158 bp.

To evaluate the effects of additives, bovine serum albumin (BSA; 019-21272, FUJIFILM Wako Pure Chemical, Osaka, Japan) was added to the PCR reaction at a final concentration of 1.1%, as described previously [17]. For comparison, dextran 40,000 (049-22331, FUJIFILM Wako Pure Chemical) was added at a final concentration of 1.1%, and Blocking One-P (05999-84, Nacalai Tesque, Kyoto, Japan) was added at a final concentration of 1.2%. All concentrations represent final percentages in the PCR reaction mixture.

### 2.3. Sample Loading into a PDMS MWA Chip and dPCR Using a Thermal Cycler

Samples were loaded into the PDMS MWA chip following the report by Hosokawa et al. [4]. First, the PDMS MWA chip (PDMS MWA chip with or without 8% MMS-003, internal hydrophilizing agent) was degassed in a vacuum chamber for at least 40 min.

Next, the dPCR PDMS MWA chip was placed on a 76 × 52 mm glass slide (S9213, Matsunami Glass, Osaka, Japan), over which 40 µL of reaction mixture was dispensed, and the assembly was left to stand for several minutes. After the sample was introduced into the microwells through capillary action, a 100 g weight was placed on top of the PDMS MWA chip for 10 min to expel the excess reaction mixture. The weight was then removed, and the residual reaction mixture around the PDMS MWA chip was wiped off using a lint-free wipe (BEMCOT™ M-1, Asahi Kasei, Tokyo Japan).

Subsequently, 20 µL of perfluorodecalin (PFD; P0837, Tokyo Chemical Industry, Tokyo, Japan) was applied to the edge of the dPCR PDMS MWA chip, and the chip was gently peeled off from the glass slide, starting at the edge. The peeled PDMS MWA chip was transferred onto a PDMS-coated glass slide that had been pre-covered with 40 µL of PFD. A 100 g weight was placed on the chip for 10 min to remove excess PFD and ensure close contact between the PDMS MWA chip and glass slide. The resulting PDMS MWA chip was then placed in a thermal cycler.

A 0.5 mm thick copper plate was mounted on the surface of the heat block of a thermal cycler (C1000 Thermal Cycler, Bio-, Hercules, CA, USA). A water holder fabricated from a 3 mm thick silicone rubber sheet (6-611-05, AS ONE, Osaka, Japan) was placed around the dPCR PDMS MWA chip. The water holder was divided into two compartments, each filled with 1.86 mL of sterile water. After filling with sterile water, a polyester film (Lumirror film T60, transparent, 50 µm-thick), a Bemcot, a glass slide, and a 35 mm Petri dish lid (6 mm thick), used as a spacer to adjust the lid height of the thermal cycler, were added.

The dPCR PDMS MWA chip containing the sealed PCR reaction mixture was then placed in a thermal cycler (C1000 Thermal Cycler, Bio-Rad), and amplification was performed. The thermal cycling protocol consisted of an initial denaturation at 96 °C for 10 min, followed by 39 cycles of two-step program comprising 98 °C for 30 s and either 56 °C or 60 °C for 2 min. After cycling, the reaction was incubated at 60 °C for 2 min, then held at 15 °C. The hot lid of the thermal cycler was turned off throughout the experiment.

### 2.4. Preparation of cDNA Templates

Human oral squamous cell carcinoma HSC4 cells (RIKEN Cell Bank, Ibaraki, Japan) were cultured in 60 mm culture. Dishes were washed once with 1 mL of phosphate-buffered saline without calcium and magnesium (PBS (−)). Total RNA was extracted by adding 1 mL of TRIzol Reagent (15596026, Thermo Fisher Scientific) to lyse the cells. The lysate was transferred to a 1.5 mL microcentrifuge tube and homogenized by passing it five times through a 21-gauge needle (Terumo non-bevel needle, Terumo, Tokyo, Japan) attached to a 1 mL syringe (Terumo).

Chloroform (200 µL; reagent grade, 034 02603, FUJIFILM Wako Pure Chemical) was added to the lysate, which was then vigorously shaken for 15 s and centrifuged at 4 °C and 12,000 rpm for 5 min using a high-speed centrifuge (TOMY Seiko, Tokyo, Japan). After phase separation, the upper aqueous phase was transferred to a new 1.5 mL tube, and 500 µL of 2-propanol (special grade, 32435-00, Kanto Chemica, Tokyo, Japan) was added, followed by thorough mixing via inversion. The mixture was incubated at room temperature for 10 min and centrifuged at 4 °C and 14,000 rpm for 10 min.

The supernatant was removed via pipetting, and the RNA pellet was washed with 800 µL of 75% ethanol. After gentle pipetting to detach the pellet from the tube wall, the sample was centrifuged again at 4 °C and 14,000 rpm for 5 min. The supernatant was discarded, and the pellet was air-dried until it became semi-transparent. The RNA pellet was dissolved in 10 µL of RNase-free water (RNeasy Mini Kit, 74104, Qiagen, Venlo, The Netherlands). RNA concentration was measured using a NanoDrop One C microvolume spectrophotometer (ND-ONEC-W, Thermo Fisher Scientific), and samples with A260/A280 ratios between 1.8 and 2.0 were used in subsequent experiments.

cDNA synthesis was performed using a PrimeScript FAST RT reagent Kit with gDNA Eraser (RR092A, Takara Bio). In a sterilized 0.2 mL tube placed on ice, 2.0 µL of 8× gDNA Eraser Premix, 2 µg of total RNA, and RNase-free water were mixed to a final volume of 16 µL and incubated at room temperature for 5 min. Subsequently, 4 µL of 5× RT Premix was added, and reverse transcription was carried out at 37 °C for 15 min. The reaction was terminated through heating at 85 °C for 5 s, followed by cooling on ice. The synthesized cDNA was stored at −20 °C until use.

### 2.5. cDNA as a dPCR Template

cDNA was diluted to a concentration of 50 ng/µL using EASY Dilution II (Takara Bio). The PCR reaction mixture was prepared by combining 1.0 µL of cDNA solution (final concentration, 1.25 ng/µL), 20 µL of QuantStudio 3D Digital PCR Master Mix v2 (A26358, Thermo Fisher Scientific), 2.8 µL each of 10 µM forward primer (Vegf-a Forward) and 10 µM reverse primer (Vegf-a Reverse), and 1.0 µL of 10 µM TaqMan probe (Vegf-a Probe). As QuantStudio™ 3D Digital PCR Master Mix v2 does not contain ROX dye, 0.8 µL of 50× ROX Dye (high ROX, KAPA Biosystems) was added to confirm the proper partitioning of the reaction mixture. RNase-free water and additives were added as needed to adjust the final reaction volume to 40 µL. The primer and probe sequences used in this study are listed in Table 1. The expected length of the PCR amplicon was 188 bp.

The additive conditions were the same as in the dPCR experiments, using plasmid DNA as the template. Specifically, BSA was added to the PCR reaction mixture to achieve a final concentration of 1.1%. For comparison, dextran was added at a final concentration of 1.1%, and Blocking One-P was added at a final concentration of 1.2%.

The thermal cycling protocol consisted of initial denaturation at 96 °C for 10 min, followed by 39 cycles of a two-step program comprising 98 °C for 30 s and 56 °C for 2 min. After cycling, the reaction was incubated at 60 °C for 2 min, then held at 15 °C. The hot lid of the thermal cycler was switched off throughout the experiment.

### 2.6. Fluorescence Microscopy and Image Acquisition

Fluorescence imaging of the PDMS MWA chip was performed using an upright fluorescence microscope (BX53, Evident, Tokyo, Japan) equipped with a CMOS camera (DP23; Evident). A 2× objective lens (PLAPON 2×, N1480200, NA = 0.08, Evident) was used for all observations. The PDMS MWA chip consisted of nine units, and each unit was imaged in a single field of view, resulting in a total of nine fields acquired per chip.

Prior to imaging, the condenser was removed from the microscope, and the light-path selector was set to 100% of the camera output. All images were acquired in grayscale and saved as TIFF files. Focus adjustment was performed using a green excitation filter cube (U-FGW; excitation 530–550 nm, emission 575 nm, dichroic mirror 570 nm) by observing the fluorescent ROX dye in the microwells. After focus adjustment, the same field of view and focal plane were maintained, and fluorescence images for quantitative analysis were acquired using a blue excitation filter cube (U-FBWA; excitation 460–495 nm, emission 510–550 nm, dichroic mirror 505 nm). The exposure time was set to 350 ms, and the fluorescence intensity was fixed at 100%. For representative images, brightness and contrast were adjusted within the range of 50–110 for display. Quantitative image analysis was performed using the acquired raw and unprocessed image data.

Image analysis was performed using ImageJ software (ver. 1.54d, National Institutes of Health, Bethesda, MD, USA). The DNA template concentration in the samples was quantified by calculating the average number of DNA copies per well (λ), assuming that DNA molecule distribution in the wells followed a Poisson distribution [18].

### 2.7. Real-Time PCR

The real-time PCR reaction mixtures were prepared to a final volume of 20 µL and dispensed into a 48-well plate. Two experimental conditions were tested: (i) no additive and (ii) 1.1% BSA added. For each condition, positive reaction mixtures containing plasmid DNA as the template and negative reaction mixtures without template DNA were prepared and analyzed in triplicates.

After dispensing, the reaction plate was sealed with MicroAmp 48-Well Optical Adhesive Film (Applied Biosystems, Waltham, MA, USA), and amplification was performed using a StepOne Real-Time PCR System (Applied Biosystems) with the TaqMan^®^ probe method (Quantitation–Standard Curve). The thermal cycling protocol consisted of an initial denaturation step at 96 °C for 10 min, followed by 39 cycles of a two-step program comprising 98 °C for 30 s and 60 °C for 2 min. After cycling, the reaction was held at 60 °C for 2 min, then at 15 °C. Fluorescence signals were acquired during the annealing/extension step at 60 °C. Data analysis was conducted using StepOne Software v2.3, and the threshold line was set uniformly at 0.2 for all conditions.

### 2.8. Statistical Analysis

All quantitative data are presented as mean ± standard deviation (SD), with *n* = 3 for all experiments. Comparisons between two groups were performed using a Welch’s *t*-test. For comparisons among three or more groups, one-way analysis of variance (ANOVA) was conducted, followed by Tukey’s post hoc multiple comparison test. A *p* value of less than 0.05 was considered statistically significant. All statistical analyses were performed using Excel Statistics (BellCurve for Excel).

## 3. Results and Discussion

### 3.1. Fabrication of PDMS MWA Chip and PCR Setup

**Figure 1:**
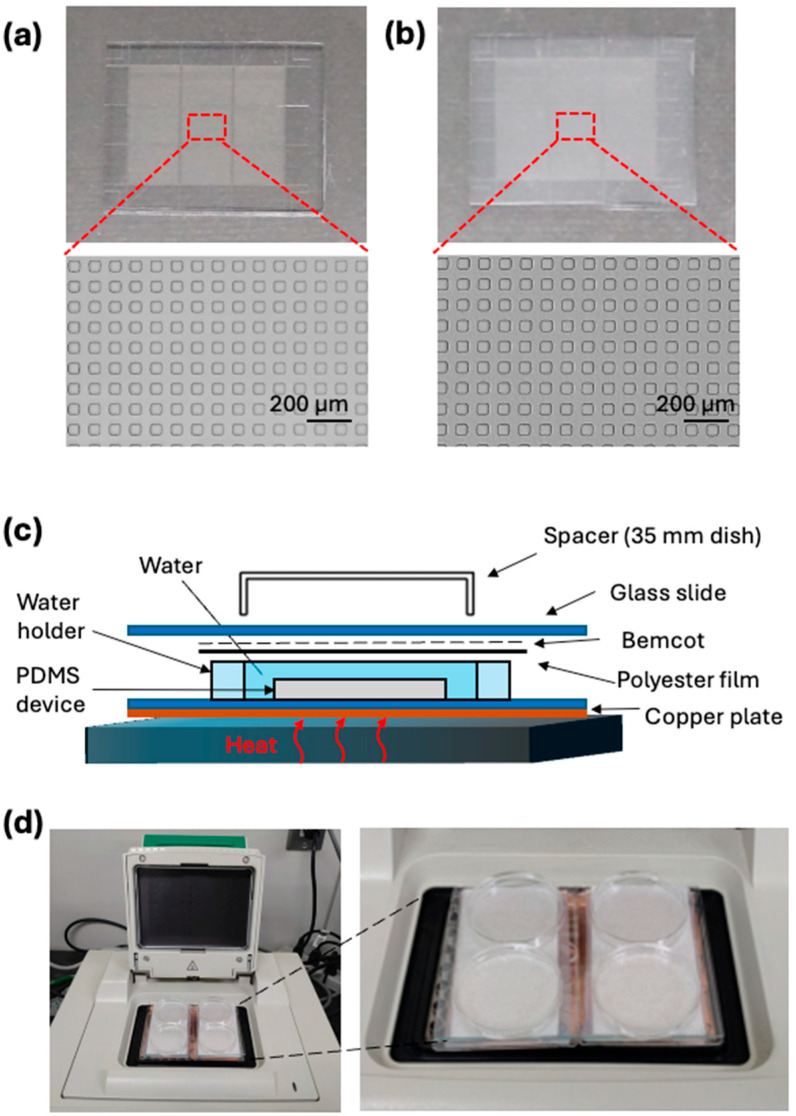
Schematic illustrations of the digital PCR (dPCR) system using a poly(dimethylsiloxane) (PDMS) microwell array (MWA) chip. (**a**) Photograph of the PDMS MWA chip. The chip dimensions were approximately 28 × 24 × 1.5 mm. (**b**) Photograph of the PDMS MWA chip with 8% crosslinking internal hydrophilizing agent MMS-003. (**c**,**d**) Setup for thermal cycling. (**c**) To prevent evaporation of the PCR mixture, the PDMS MWA chip was immersed in water in a water holder fabricated from a silicone rubber plate. The water holder was covered with a polyester film, where a glass slide and spacer were placed. (**d**) Photograph of the PDMS MWA chip positioned on the thermal cycler.

Figure 1a,b show the PDMS MWA chips fabricated in this study. The chips prepared without (Figure 1a) and with a crosslinking internal PDMS hydrophilizing agent (Figure 1b) exhibited comparable microstructure geometries, indicating that the intended chip design was reproducibly achieved under both conditions.

As illustrated in Figure 1c, the fabricated PDMS chip was placed on a copper plate and assembled for thermal contact, and the assembled structure was set into a PCR instrument for amplification (Figure 1d). This configuration enabled stable PCR operation using the PDMS-based chip and was used in all subsequent experiments.

### 3.2. dPCR Using a Plasmid Template (PDMS MWA Chip)

dPCR was performed on a PDMS MWA chip without an internal hydrophilizing agent, and BSA, Blocking One-P, or dextran were added to the reaction mixture to compare their effects on quantification. BSA and Blocking One-P function as blocking agents, whereas dextran is not expected to exert a blocking effect, but is known as a macromolecular crowding agent that can promote enzymatic reactions [19].

**Figure 2:**
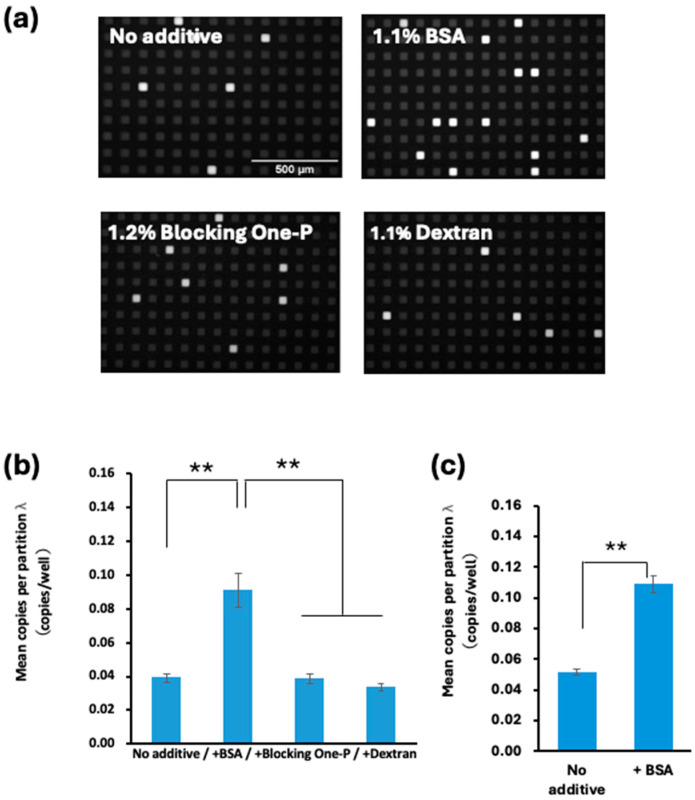
dPCR results using plasmid template. (**a**) Fluorescence microscopy image of dPCR wells obtained on a PDMS MWA chip without a crosslinking internal hydrophilizing agent. The reaction mixture contained 1.1% BSA, 1.2% Blocking One-P, and 1.1% dextran. (**b**) Bar graph comparing dPCR quantification under four additive conditions—no additive, BSA, Blocking One-P, and dextran—measured on a PDMS MWA chip without an internal hydrophilizing agent. Means ± SD, one-way ANOVA, **: *p* < 0.01, *n* = 3. (**c**) Bar graph showing the effect of BSA on dPCR quantification using a PDMS MWA chip containing 8% of the crosslinking internal hydrophilizing agent MMS-003. Means ± SD, one-way ANOVA, **: *p* < 0.01, *n* = 3.

Reactions proceeded under all conditions: no additive, 1.1% BSA, 1.2% Blocking One-P, and 1.1% dextran (Figure 2a). The background fluorescence level (grayscale ~55) and blank signal (~60) were clearly distinguishable from the positive signal (~100), allowing robust thresholding and reliable discrimination between positive and negative microwells. Figure 2b shows the quantification results for each condition tested. The average DNA copy number obtained via dPCR was highest with 1.1% BSA, at 0.091 ± 0.010 copies/well (*n* = 3), which was significantly greater than the values for the other conditions (*p* < 0.01). In contrast, the values for the no additive condition (0.039 ± 0.002 copies/well; *n* = 3), 1.2% Blocking One-P (0.039 ± 0.003 copies/well; *n* = 3), and 1.1% dextran (0.034 ± 0.002 copies/well; *n* = 3) did not differ appreciably, indicating no notable improvement with 1.2% Blocking One-P or 1.1% dextran compared with no additive.

In this study, despite using the same amount of DNA template, the average DNA copy number differed between the conditions. This suggests that because the PDMS microwell is highly hydrophobic, the DNA template, primers, and polymerase may have adsorbed onto the well walls, thereby reducing their effective concentrations in the reaction mixture. This loss of effective reactants likely decreased positive well formation rate, leading to an apparent reduction in the average copy number.

In contrast, the increased average DNA copy number observed under BSA supplementation suggests that BSA preferentially adsorbed onto the PDMS surface and suppressed the nonspecific adsorption of the DNA template and enzymes. In addition, BSA may have contributed to the stabilization of polymerase activity during the reaction.

Notably, Blocking One-P is primarily designed to suppress nonspecific adsorption on conventional solid substrates with a high protein-binding capacity, such as ELISA microplates and Western blot membranes. Because the physical adsorption and functional stability of polymeric blocking layers strongly depend on substrate surface chemistry, highly hydrophobic surfaces, such as untreated PDMS, are generally unfavorable for forming thermodynamically stable and uniform polymer adsorption layers [20]. Consequently, the blocking layer formed by this reagent on untreated PDMS surfaces may exhibit limited stability and effectiveness.

### 3.3. Evaluation of a PDMS MWA Chip Containing MMS-003 for dPCR

dPCR was performed on a PDMS MWA chip containing 8% MMS-003, a crosslinking internal PDMS hydrophilizing agent. According to the manufacturer’s specifications, the static water contact angle at 5 min is approximately 100° for standard PDMS and approximately 45° for hydrophilized PDMS containing 8% MMS-003 (for reference, glass typically exhibits a contact angle of 0–30° [21]). In this study, we examined whether an internal PDMS hydrophilizing agent would suppress enzyme and DNA template adsorption to the well walls, thereby increasing the average DNA copy number.

In the evaluation using standard PDMS chips, we compared the conditions with and without BSA. Under BSA-free conditions, the average was 0.052 ± 0.001 copies/well (*n* = 3), compared to 0.109 ± 0.003 copies/well under 1.1% BSA (*n* = 3). A statistically significant difference was observed between the two conditions (*p* < 0.01, Figure 2c). This difference is consistent with the trend observed for the PDMS MWA chip without an internal hydrophilizing agent, where BSA addition increased the average copy number.

In contrast, comparing BSA-free conditions between PDMS MWA chips without an internal hydrophilizing agent (Figure 2b) and those containing 8% MMS-003 (Figure 2c), the averages were 0.039 ± 0.002 and 0.052 ± 0.001 copies/well, respectively. Under the present conditions, hydrophilization with MMS-003 alone did not lead to a statistically significant increase in the average copy number. According to the manufacturer, PDMS modified with MMS-003 is suitable for cell culture. While the surface is thought to form an interface with weak protein interactions, it is not fully resistant to nonspecific adsorption, and therefore its ability to suppress it is limited. As a result, it did not provide a sufficient improvement to increase the average copy number. Based on these results, the remaining experiments in this work were conducted using PDMS MWA chip without an internal hydrophilizing agent.

### 3.4. Effect of BSA on dPCR Quantification Using a Plasmid Dilution Series

**Figure 3:**
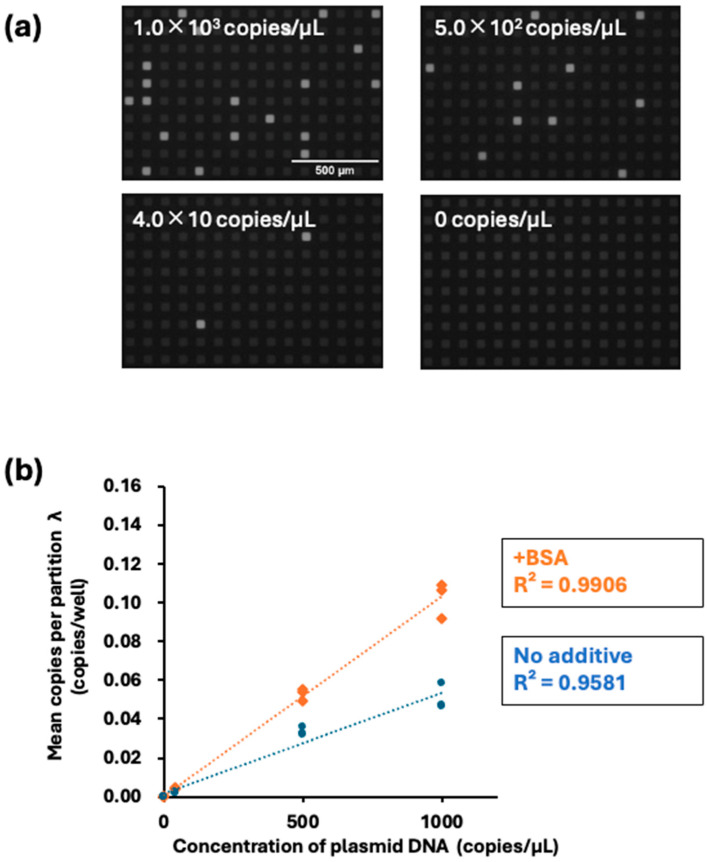
dPCR was performed using serially diluted plasmid template. (**a**) Fluorescence microscopy images of dPCR microwells acquired at four plasmid concentrations under the condition of 1.1% BSA addition. The number of positive microwells increased along with the input plasmid concentration. (**b**) Quantitative plots of average DNA copy number versus plasmid concentration under BSA-added (orange diamonds) and BSA-free (blue circles) conditions. Linear fits yielded slopes of *m* = 1.0 × 10^−4^ (BSA-added) and *m* = 0.5 × 10^−4^ (BSA-free), with coefficients of determination of *R*^2^ = 0.9906 and *R*^2^ = 0.9581, respectively.

Next, we performed dPCR using a four-point series of plasmid-template concentrations. Representative fluorescence microscopy images of the chips are shown in Figure 3a. The number of positive microwells increased along with the plasmid concentration. The corresponding quantitative results are shown in Figure 3b. A good linear relationship was observed with plasmid concentration; the slope of the linear fit was *m* = 1.0 × 10^−4^ for BSA-added conditions and *m* = 5.0 × 10^−5^ for BSA-free conditions. Thus, BSA-added conditions yielded a higher average DNA copy number, indicating improved quantitative sensitivity. The coefficients of determination were *R*^2^ = 0.9581 (BSA-free) and *R*^2^ = 0.9906 (BSA-added), demonstrating higher linearity with BSA.

### 3.5. Effect of BSA on DNA Quantification Using Conventional PCR Tubes and Real-Time PCR

In dPCR, the average DNA copy number was higher under BSA-added than under BSA-free conditions, and a statistically significant difference was observed (Figure 2b and Figure 3b). To determine whether this effect could be attributed to surface interactions specific to the PDMS MWA chip, or it represented a general phenomenon in PCR, real-time PCR was performed using conventional PCR tubes made of polypropylene, and Ct values were compared between BSA-free and 1.1% BSA conditions (Figure S1). The Ct value was 26.672 ± 0.025 in the absence of BSA and 26.593 ± 0.030 in the presence of BSA (*n* = 3). Although the difference between the two conditions was small (approximately 0.08 Ct), this corresponds to an approximately 1.057-fold increase in copy number in the presence of BSA. Welch’s *t*-test indicated a significant difference (*p* = 0.026).

However, compared with the pronounced signal enhancement observed in dPCR performed using the PDMS MWA chip, the difference observed in real-time PCR was minimal, suggesting a limited effect of BSA under these conditions. These results suggest that the primary effect of BSA is not to substantially enhance the PCR reaction itself, but rather to suppress the nonspecific adsorption of DNA and/or enzymes to the PDMS well surface, given the minimal impact of PCR tubes. In other words, the effect of BSA is not evident in real-time PCR performed in conventional PCR tubes, where surface adsorption effects are relatively small. However, its impact becomes pronounced in dPCR conducted in a PDMS MWA chip with a large surface area-to-volume ratio and hydrophobic surfaces.

### 3.6. dPCR Using Cell-Derived cDNA

dPCR quantification was performed using cDNA derived from HSC4 cells as the DNA template, employing a TaqMan probe–based assay. As in the experiments using plasmid DNA as the template, we examined whether the addition of BSA, Blocking One-P, or dextran altered the average DNA copy number.

**Figure 4:**
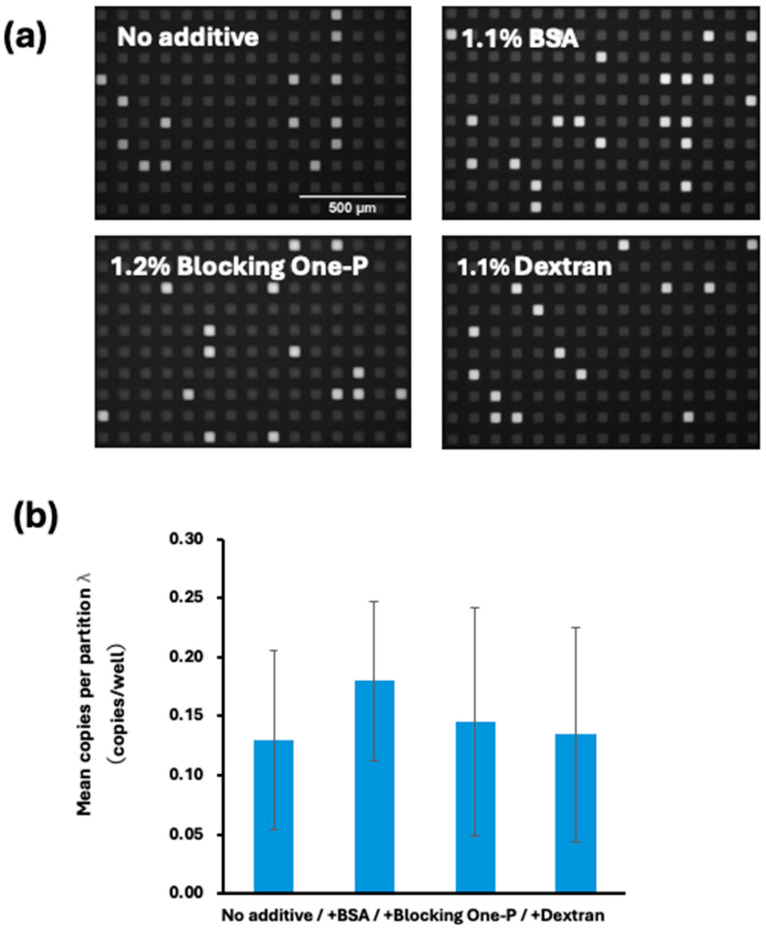
dPCR results using cDNA from HSC4 cells. (**a**) Fluorescence microscopy image of dPCR wells obtained using a PDMS MWA chip. The reaction mixture contained 1.1% BSA, 1.2% Blocking One-P, and 1.1% dextran. (**b**) Bar graph comparing dPCR quantification under four additive conditions (no additive, BSA, Blocking One-P, and dextran) measured on a PDMS MWA chip. Means ± SD, one-way ANOVA, *n* = 3.

As shown in the fluorescence images in Figure 4a, amplification signals were observed under all conditions tested, including no additive, 1.1% BSA, 1.2% Blocking One-P, and 1.1% dextran. The average DNA copy numbers were 0.13 ± 0.08 copies/well (*n* = 3) for the no-additive condition, 0.18 ± 0.07 copies/well (*n* = 3) with 1.1% BSA, 0.15 ± 0.10 copies/well (*n* = 3) with 1.2% Blocking One-P, and 0.13 ± 0.09 copies/well (*n* = 3) with 1.1% dextran (Figure 4b).

In the dPCR quantification using cDNA as the template, no statistically significant differences were detected between the conditions (one-way ANOVA, *p* ≈ 0.9 > 0.05). Compared with the results obtained using plasmid DNA as the template (Figure 2b), a generally larger variability was observed. Because the same cDNA sample was used as the template for all conditions, the increased variability is unlikely to originate from the reverse transcription reaction itself, but may instead be partially attributed to heterogeneity in cDNA molecular integrity and length. In addition, residual protein components may be present in cDNA samples, which could reduce the relative effectiveness of BSA in suppressing surface adsorption, thereby making its impact less apparent in cDNA-based dPCR assays.

### 3.7. Effect of BSA on dPCR Quantification Using a cDNA Dilution Series

**Figure 5:**
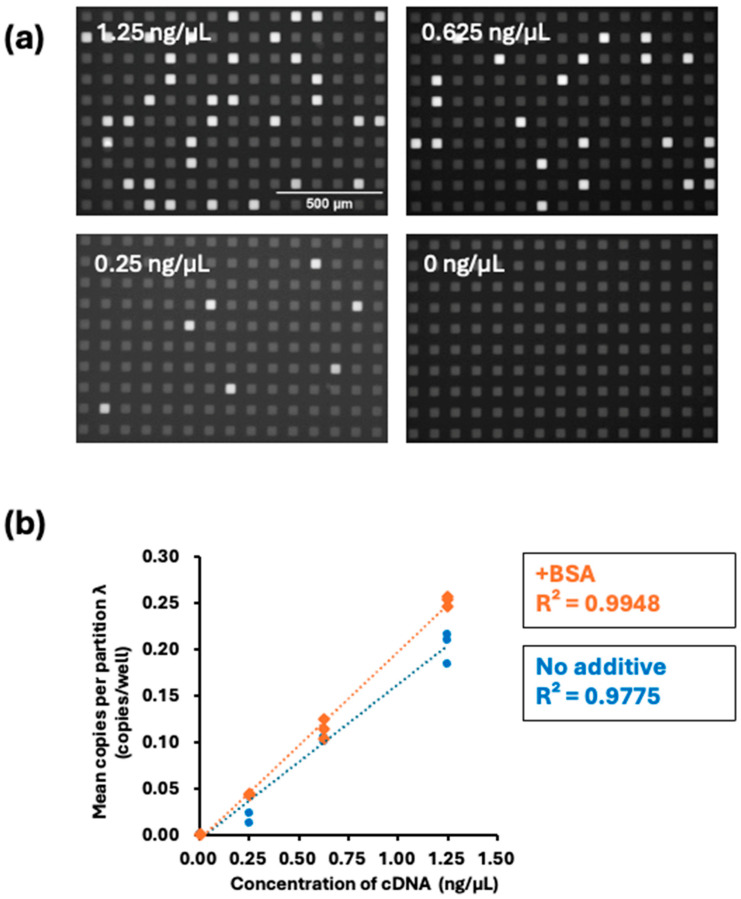
dPCR was performed using serially diluted cDNA templates. (**a**) Fluorescence microscopy images of dPCR wells on a PDMS MWA chip using serially diluted cDNA from HSC4 cells. (**b**) Quantitative plots of average DNA copy number versus varying cDNA concentrations under BSA-added (orange diamonds) and BSA-free (blue circles) conditions. Linear fits yielded slopes of *m* = 0.20 (BSA-added) and *m* = 0.17 (BSA-free), with coefficients of determination *R*^2^ = 0.9948 and *R*^2^ = 0.9775, respectively.

To further evaluate the effect of 1.1% BSA on dPCR quantification, measurements were performed using four different cDNA concentrations as the template, and the results obtained with and without BSA were compared. As shown in the fluorescence images in Figure 5a (BSA-added reaction mixture), the number of positive microwells increased along with cDNA concentrations. In contrast, no positive wells were observed in negative control reactions.

When the average DNA copy number was plotted against the four cDNA concentrations, a linear relationship dependent on the cDNA concentration was observed under both conditions (Figure 5b). Linear regression analysis yielded slopes of *m* = 0.20 and *m* = 0.17 for the BSA-added and BSA-free conditions, respectively. The former showed slightly higher average DNA copy numbers. The coefficients of determination were *R*^2^ = 0.9775 in the absence of BSA and *R*^2^ = 0.9948 in the presence of BSA, indicating a slightly higher linearity under BSA-added conditions.

### 3.8. Limitations and Future Perspectives

PDMS-related adsorption has been recognized as a general concern in microfluidic PCR; however, its practical impact varies depending on chip architecture and operational mode. In particular, its influence on quantitative readouts has not been sufficiently explored in chamber-based dPCR systems employing sealed microwells and conventional thermal cyclers. The present study demonstrates that solution-phase blocking additives, such as BSA, can influence absolute copy number quantification even in simplified chamber-based dPCR platforms.

Nevertheless, several limitations of this study should be acknowledged. In the present study, experiments were conducted not only using plasmid DNA but also using RNA extracted from cells, and similar trends were observed. These results support the notion that the effects of BSA are reproducible under conditions closer to biological samples. However, when RNA is used as a template, additional factors such as the efficiency and variability of reverse transcription, as well as the presence of sample-derived impurities, may influence the quantification results. Moreover, the range of sample conditions examined in this study was limited, and further validation using different cell types and more complex biological samples is required.

In addition, although the observed effect of BSA is presumed to be primarily due to suppression of nonspecific adsorption to PDMS surfaces, direct measurements of adsorption or detailed surface analyses were not performed. Therefore, the underlying mechanism has not been fully elucidated. Furthermore, although several types of additives were examined in this study, the range of additive concentrations and conditions investigated remained limited. Further optimization of these parameters, as well as systematic comparison with a broader range of blocking agents, will be necessary in future studies. Together, these findings provide a basis for further refinement of chamber-based dPCR systems through both material and solution-level optimization.

## 4. Conclusions

In this study, we demonstrated that solution-phase blocking additives can significantly influence absolute copy number quantification in chamber-based digital PCR systems. The addition of BSA increased the measured DNA copy number, indicating that suppression of nonspecific adsorption to PDMS surfaces is a key factor contributing to improved quantification accuracy.

This effect was not observed in conventional real-time PCR using polypropylene tubes, suggesting that the influence of BSA is primarily observed in PDMS-based microfluidic environments with high surface-to-volume ratios.

These findings highlight that solution-phase additives play a critical role in determining quantification outcomes in dPCR platforms. Therefore, careful consideration of surface interactions and additive conditions is essential for accurate absolute quantification in PDMS-based systems. This study provides a practical basis for further improvement of chamber-based dPCR technologies.

## Figures and Tables

**Table 1:** Primer and Probe Sequences.

	Sequence (5′→3′)
NIID_2019-nCoV_N_F2	AAATTTTGGGGACCAGGAAC
NIID_2019-nCoV_N_R2	TGGCAGCTGTGTAGGTCAAC
NIID_2019-nCoV_N_P2	FAM-ATGTCGCGCATTGGCATGGA-BHQ
Vegf-A_Forward	AGACGGACAGAAAGACAG
Vegf-A_Reverse	AAGCAGGTGAGAGTAAGC
Vegf-A_Probe	FAM-CCAAAGCACAGCAATGTCCTGAAGC-TAM

FAM (6-carboxyfluorescein), BHQ (Black Hole Quencher), and TAM (tetramethylrhodamine).

## Data Availability

The original contributions presented in this study are included in the article/Supplementary Material. Further inquiries can be directed to the corresponding author.

## Supplementary Materials

The following supporting information can be downloaded at: https://www.mdpi.com/article/10.3390/mi17070791/s1, Figure S1: Amplification plots from real-time qPCR, showing ΔRn as a function of cycle number.
