# Supplementary material for: Bovine Serum Albumin Enhances the Quantitative Performance of Polydimethylsiloxane-Based Chamber Digital PCR by Suppressing Surface Adsorption
Source: Micromachines (Basel). 2026 Jun 28;17(7):791. doi: 10.3390/mi17070791 (PMC13413812; doi:10.3390/mi17070791)
Supplement: Supplementary file 1 [file micromachines-17-00791-s001.zip › FigureS1.pptx]

## Slide 1
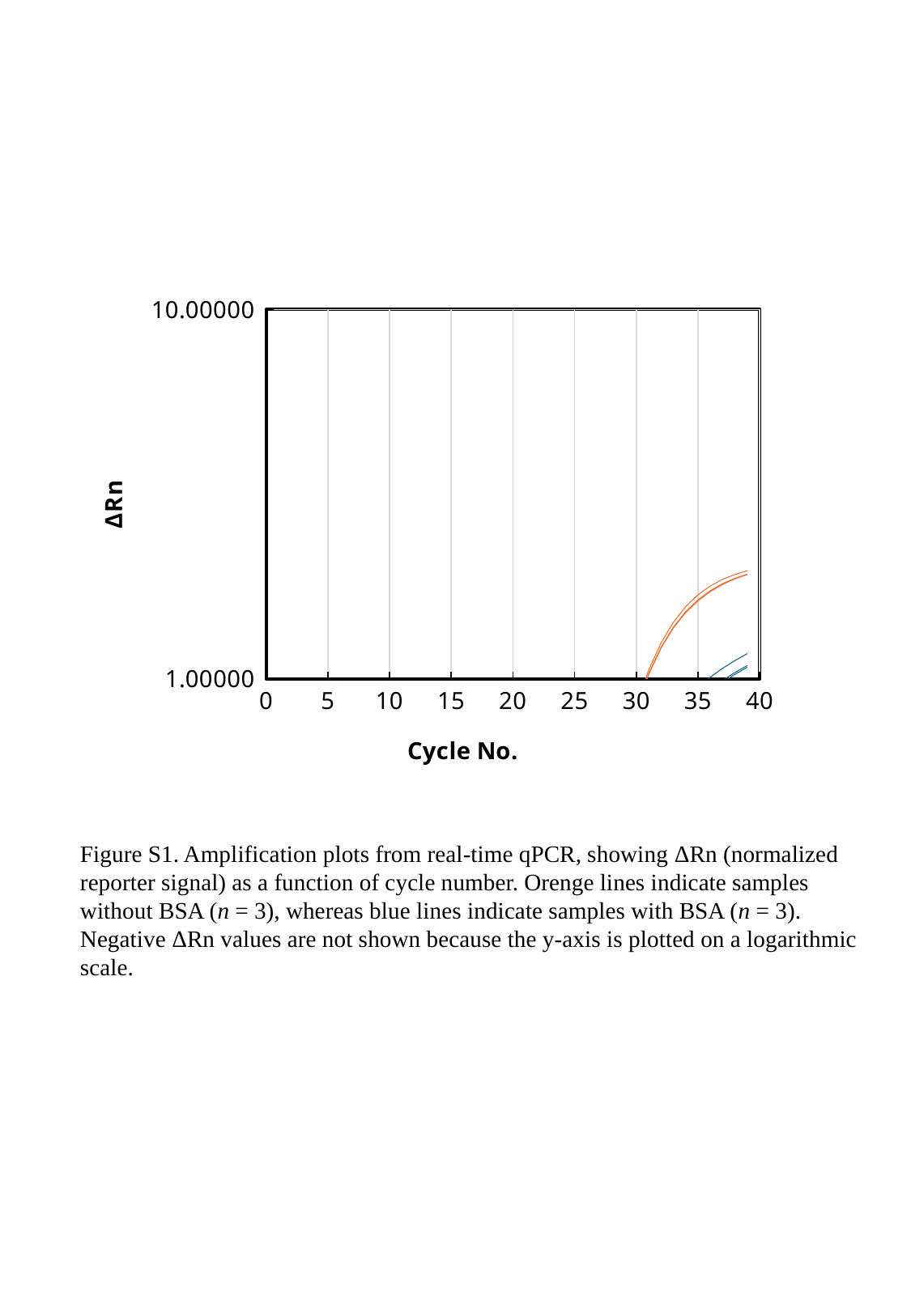

### Chart
| Category | | | | | | |
|---|---|---|---|---|---|---|Figure S1. Amplification plots from real-time qPCR, showing ΔRn (normalized reporter signal) as a function of cycle number. Orenge lines indicate samples without BSA (n = 3), whereas blue lines indicate samples with BSA (n = 3). Negative ΔRn values are not shown because the y-axis is plotted on a logarithmic scale.
